# An in vitro model for postoperative cranial nerve dysfunction and a proposed method of rehabilitation with *N*-acetylcysteine microparticles

**DOI:** 10.1007/s00405-024-08622-z

**Published:** 2024-04-23

**Authors:** Ashley Kita, Katherine Kedeshian, Michelle Hong, Larry Hoffman

**Affiliations:** 1 Department of Head and Neck Surgery, David Geffen School of Medicine at University of California Los Angeles, 10883 Le Conte Avenue, CHS 63-170, Los Angeles, CA 90095 USA; 2 Vestibular Neuroscience Laboratory, Brain Research Institute, David Geffen School of Medicine at University of California Los Angeles, Los Angeles, CA USA

**Keywords:** Neurapraxia, Axonotmesis, Nerve injury, Schwann cell, Drug delivery, Microparticles

## Abstract

**Purpose:**

When operating near cranial motor nerves, transient postoperative weakness of target muscles lasting weeks to months is often observed. As nerves are typically intact at a procedure’s completion, paresis is hypothesized to result from a combination of neurapraxia and axonotmesis. As both neurapraxia and axonotmesis involve Schwann cell injury and require remyelination, we developed an in vitro RSC96 Schwann cell model of injury using hydrogen peroxide (H_2_O_2_) to induce oxidative stress and investigated the efficacy of candidate therapeutic agents to promote RSC96 viability. As a first step in developing a long-term local administration strategy, the most promising of these agents was incorporated into sustained-release microparticles and investigated for bioactivity using this assay.

**Methods:**

The concentration of H_2_O_2_ which reduced viability by 50% was determined to establish a standard for inducing oxidative stress in RSC96 cultures. Fresh cultures were then co-dosed with H_2_O_2_ and the potential therapeutics melatonin, *N*-acetylcysteine, resveratrol, and 4-aminopyridine. Schwann cell viability was evaluated and the most efficacious agent, *N*-acetylcysteine, was encapsulated into microparticles. Eluted samples of *N*-acetylcysteine from microparticles was evaluated for retained bioactivity.

**Results:**

100 µM *N*-acetylcysteine improved the viability of Schwann cells dosed with H_2_O_2_. 100 µM Microparticle-eluted *N*-acetylcysteine also enhanced Schwann cell viability.

**Conclusion:**

We developed a Schwann cell culture model of iatrogenic nerve injury and used this to identify *N*-acetylcysteine as an agent to promote recovery. *N*-acetylcysteine was packaged into microparticles and demonstrated promise as a locally administrable agent to reduce oxidative stress in Schwann cells.

## Introduction

Surgical procedures conducted in the vicinity of peripheral nerves pose risks of injury to those nerves through iatrogenic factors such as compression or manipulation. While typically transient in nature, postoperative nerve dysfunction of cranial nerves can have devastating effects such as dysphagia, dyspnea, and facial paresis that affect individuals for weeks to months as they recover [1, 2]. The recurrent laryngeal nerve has been found to have reduced function postoperatively in up to 13% of thyroidectomy procedures, and the facial nerve was found to be paretic in up to 63% of parotidectomy surgeries [1, 3, 4]. While surgeons counsel their patients regarding these risks, there is a need for intraoperative therapeutics to protect at-risk nerves and minimize this period of functional disability. The development of a local method of delivering a therapeutic agent to the site of nerve injury during surgery, such as through focused injection through a catheter, could expedite nerve recovery and preclude systemic therapies such as steroids, which are often utilized when a nerve is found to be weak postoperatively [5, 6]. Another desirable feature of a local therapeutic is sustained release of medication through drug-eluting biocompatible polymers, similar to that achieved by steroid-releasing sinus stents [7]. One strategy for sustained release involves blending medications into small particles of dissolvable polymers which can be injected as a solution or gel. While different size particles have been explored for head and neck applications, microparticles offer the advantage of injectability for focused delivery and avoid the cellular uptake and toxicity seen with smaller particles [8, 9].

In designing a therapeutic for inadvertent operative nerve injury it is important to understand the pathophysiology of nerve injury to promote rehabilitation. Seddon first proposed a system of classifying nerve injuries wherein he described three levels of nerve injury: neurapraxia, axonotmesis, and neurotmesis [10]. Sunderland expanded this to five categories based on histopathology and associated clinical and findings [11]. Neurapraxia was described as a Grade I injury involving temporary interruption of the myelin sheath without loss of axon continuity. Axonotmesis was expanded to grade II–IV injuries describing a loss of continuity of the axon and its myelin sheath with varying degrees of injury to the neural connective tissue just short of complete transection (Grade V). Recovery from axonotmesis takes considerable time and is guided by the intact connective tissue scaffold of the nerve [12]. Given the duration of paresis after operations near cranial nerves, it is reasonable to consider this a form of injury ranging from neurapraxia to axonotmesis. Thus, efforts to shorten this period of dysfunction should promote both axonal and Schwann cell rehabilitation. As Grade I through IV injuries all involve disruption of myelination, promoting Schwann cell viability to support remyelination may be an effective strategy to reduce the period of functional disability after any procedure where there is concern that manipulation may have resulted in nerve injury.

In vitro cell culture models can be helpful in identifying therapeutics to promote Schwann cell viability. The RSC96 rat primary Schwann cell line is a common model for investigating therapeutic agents to ameliorate crush injuries [13]. A common factor among nerve injuries is oxidative stress, and therefore hydrogen peroxide (H_2_O_2_) has been used as a source of oxygen-derived free radicals to mimic such injuries [14–16]. In the present study, H_2_O_2_-treated RSC96 cultures were used as a model of Schwann cell compromise to screen four therapeutic agents for their ability to promote Schwann cell health: melatonin, *N*-acetylcysteine, resveratrol, and 4-aminopyridine. Melatonin is a hormone thought to promote Schwann cell rehabilitation by detoxifying free radicals via electron donation [17–19]. *N*-acetylcysteine (NAC) is an antioxidant that promotes cell viability [20]. Resveratrol is an antioxidant thought to promote autophagy and improve recovery after Wallerian degeneration [21]. And 4-aminopyridine is a non-selective potassium channel blocker used to treat demyelinating disorders [22]. While the neuroprotective effects of these agents have been previously investigated, assessment of their impact on Schwann cell viability in the presence of an oxidative stressor and suitability for a local drug delivery application were unknown.

How a therapeutic agent is administered to an injury site is an important consideration when delivering drugs locally. Following an initial screen, the most efficacious candidate therapeutic was encapsulated in biocompatible polymer microparticles as a preclinical step toward the development of a therapeutic strategy that could be deployed via targeted injection during surgery to mitigate local nerve injury. The development of such a therapeutic could also have broader prophylactic and rehabilitative implications for myelinated nerve injuries beyond cranial nerves.

## Methods

### Cell culture and reagents

RSC96 rat Schwann cells (RRID: CVCL_4694) were cultured in Dulbecco’s modified Eagle’s medium with 10% fetal bovine serum (FBS) and 1% penicillin–streptomycin, and maintained in an incubator at 37 °C and 5% CO_2_. Early passages of excess cells were stored at − 200 °C in liquid nitrogen. All reagents and materials used in the conduct of this investigation are listed in Table 1.

**Table 1:** Materials and reagents

Item	Purpose	Product ID	Source
RSC96 rat Schwann cells	Cell culture model	CRL-2756	ATCC (Manassas, Va)
Dulbecco’s modified Eagle’s media	Cell culture model	21063045	ThermoFisher Scientific (Waltham, MA)
Fetal bovine serum	Cell culture model	26140079	ThermoFisher Scientific (Waltham, MA)
Penicillin–streptomycin	Cell culture model	15140122	ThermoFisher Scientific (Waltham, MA)
Hydrogen peroxide	Injury agent	88597	Sigma–Aldrich (Saint Louis, MO)
Melatonin	Agent screened	J62452-06	Alfa Aesar (Ward Hill, MA)
*N*-Acetylcysteine	Agent screened	A7250	Sigma–Aldrich (Saint Louis, MO)
4-Aminopyridine	Agent screened	275875	Sigma–Aldrich (Saint Louis, MO)
Resveratrol	Agent screened	R5010	Sigma–Aldrich (Saint Louis, MO)
CCK-8	Cell viability assay	96992	Sigma–Aldrich (Saint Louis, MO)
Polycaprolactone	Biocompatible polymer for microparticle fabrication	L14E	Viatel (Covington, KY)

### Cell counting kit-8 viability assay

Viability of cells as an assessment of both cell number and cellular metabolic activity was determined with the cell counting kit-8 (CCK-8). Implementation of this kit provided a method by which cellular dehydrogenases (i.e. proportional to cell numbers and/or viability in culture) reduced a tetrazolium salt resulting in production of a formazan dye that was calorimetrically quantified by measuring absorbance with a plate reader at 450 nm.

### Developing a reproducible H_2_O_2_ cell culture model of oxidative stress

RSC96 cells were seeded at a density of 4000 cells/well of a 96-well plate and allowed to grow overnight. After a media change, cells were dosed with varying concentrations (0–150 µM) of H_2_O_2_. Cell viability was assessed 72 h after plating via the CCK-8 assay. A four-parameter logistic regression based upon two replicate experiments (*n* = 8 each) was used to estimate the H_2_O_2_ concentration resulting in a 50% reduction of RSC96 viability, referred to as the LC_50_. This dose was used in all subsequent trials to induce RSC96 injury.

### Screening potentially therapeutic agents

The protective efficacies of potential therapeutic agents were assessed in RSC96 cells plated at a density of 4000 cells/well. After 24 h the media was replaced with a range of concentrations for each therapeutic agent both without and simultaneously with 60 µM of H_2_O_2_, the concentration of H_2_O_2_ resulting in 50% viability reduction (i.e. LC_50_). Cells were dosed with 0, 0.1, 1, 10, and 100 µM of melatonin, *N*-acetylcysteine (NAC), resveratrol, and 4-aminopyridine. Viability was determined with the CCK-8 assay and absorbances were normalized to their respective 0 µM condition. Melatonin was administered in serum free media to avoid the potential impact of endogenous melatonin that may be present in variable amounts in FBS lots. Representative micrographs of cultures were obtained at 10 × using a Zeiss Axio observer microscope.

### Microparticle fabrication and characterization

The screening experiments described above identified the most promising therapeutic agent, NAC, used in the balance of this research to develop a microparticle-mediated delivery system. Polycaprolactone (PCL) microparticles were fabricated with and without 10 mg NAC according to a previously established protocol from our laboratory [23–25].

### Bioactivity assessment of eluted NAC

RSC96 cells were plated in 96-well plates at a density of 4000 cells/well for 24 h and then co-dosed with H_2_O_2_ and resuspended NAC or NAC from the highest concentration eluted solution from PCL NAC microparticles, 1752 µM. As NAC was eluted into PBS, eluted solutions were diluted with media to the appropriate concentration for dosing. No more than 15% of the volume dosed contained eluted NAC solution to minimize any effects of media deprivation. The CCK-8 assay was then used to determine cell viability.

### Statistical analysis

Data analysis was performed using R Studio (R Core Team, 2017). LC_50_ was determined using a four-parameter logistic regression. One-way analyses of variance (ANOVA) with Tukey post hoc tests were used to compare therapeutic agents to control and therapeutic agents co-dosed with H_2_O_2_ to cells dosed with H_2_O_2_ alone in the therapeutic agent screening assay. One-way ANOVA was performed given different numbers of observations in the screening experiments with and without H_2_O_2_. Two-way ANOVA with Tukey post hoc analysis was used to compare the cell viability of resuspended NAC and NAC eluted from PCL microparticles when co-dosed with H_2_O_2_. Figures were created with Igor Pro (WaveMetrics, 2022) and Adobe Illustrator (Adobe Inc, 2019).

## Results

### LC_50_ determination and therapeutic agent screening

**Fig. 1:**
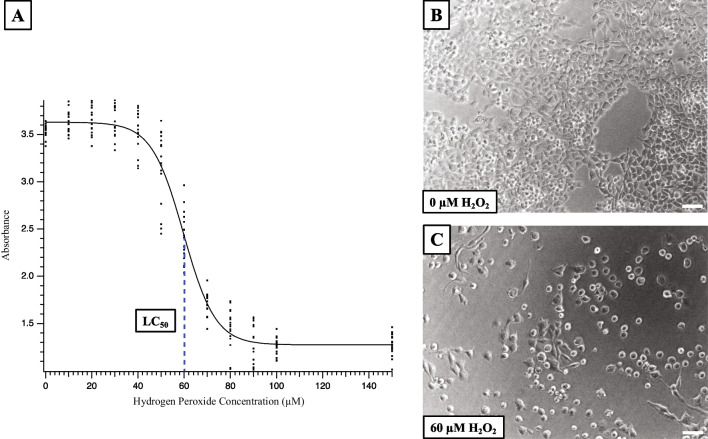
RSC96 viability at H_2_O_2_ concentrations ranging 0–150 µM as determined by CCK-8 assay. **A** H_2_O_2_ dose versus viability data fit with a four-parameter logistic regression. The data include 16 absorbances per H_2_O_2_ dose across two experiments (8 absorbance measures for each dose and experiment). **B**, **C** Light micrographs of RSC96 cells (10X) without H_2_O_2_ (**A**) and with 60 µM H_2_O_2_ for 48 h (**B**). Scale bars: 50 µM. The mean calculated dose required to reduce absorbance by 50% for the two experiments was 59.7 µM as indicated by a dashed line and LC50 label

The impact of H_2_O_2_ on RSC96 cultures is shown in Fig. 1. The micrographs represent RSC96 cells 48 h after original plating at a density of 4000 cells, and 24 h after a media change only (Fig. 1B) or a media change with 60 µM H_2_O_2_ (Fig. 1C). The cells in Fig. 1C clearly exhibited reduced density reflecting the negative impact on cell viability induced by H_2_O_2_. This impact was quantified by measuring absorbance resulting from the CCK-8 assay over a broad range of H_2_O_2_ concentrations (Fig. 1A) for two independent experiments in which absorbances were measured at each concentration (*n* = 8/concentration/experiment). These data were fit by four-parameter logistic regression (solid line), resulting in LC50 estimates of 59.0 and 60.3 µM for each experiment. The mean LC_50_ value for both experiments of 59.7 µM was rounded to 60 µM for simplicity in all subsequent experiments.

**Fig. 2:**
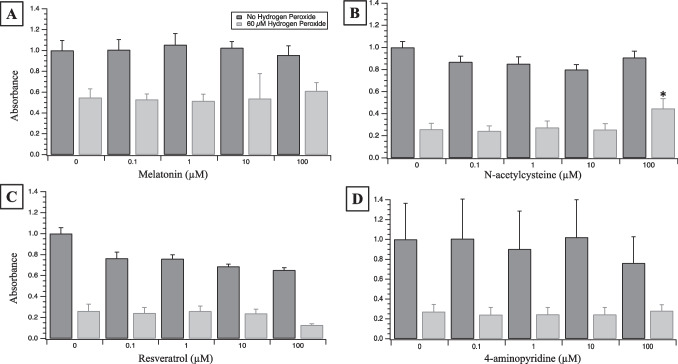
**A**–**D** Screening four therapeutic agents for their potential to improve RSC96 culture viability when dosed alone and with 60 µM H_2_O_2_. Dark grey bars reflect normalized absorbance measures (see “Results” text) of RSC96 cells dosed over three orders of concentration magnitude for each therapeutic agent. Light gray bars reflect CCK-8 assay absorbances of RSC96 cells co-dosed with 60 µM hydrogen peroxide and each agent. Only NAC at 100 µM exhibited a protective impact from viability compromise caused by 60 µM H_2_O_2_ alone (*: *p* < 0.05)

The 60 µM H_2_O_2_ dose was used to screen four potentially therapeutic agents: melatonin, NAC, resveratrol, and 4-aminopyridine. Figure 2 illustrates the impact of agent concentration on RSC96 culture viability applied 24 h after original plating and measured with the CCK-8 assay another 48 h after administration. The absorbance values obtained for melatonin and 4-aminopyridine across all concentrations exhibited no increase to support a conclusion of enhancement of viability (*p* = 0.099 and *p* = 0.63 respectively). Resveratrol not only showed no increase in viability, but showed a decrease in viability at 100 µM (*p* < 1 × 10^–6^). Conversely, cells co-dosed with 60 µM of H_2_O_2_ and 100 µM NAC demonstrated an increased viability compared to the H_2_O_2_-alone condition (0 µM NAC) or lower NAC concentrations (concentration main effect: *p* < 2 × 10^–16^; H_2_O_2_ main effect: of *p* < 2 × 10^–16^; interaction effect: *p* < 2 × 10^–16^). This supported further exploration of the therapeutic efficacy of 100 µM NAC in this RSC96 model of Schwann cells.

### Bioactivity of eluted NAC

**Fig. 3:**
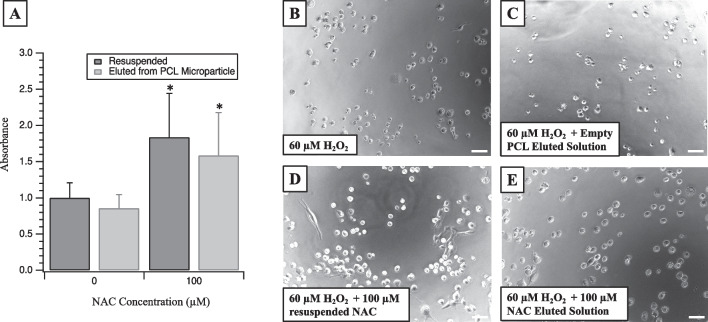
NAC eluted from PCL microparticles has similar effects as freshly resuspended NAC in the presence of 60 µM hydrogen peroxide. **A** RSC96 cell viability as measured by CCK-8 assay demonstrating increased viability when dosed with resuspended or eluted NAC (*p* = 8.9 × 10^–12^). **B**–**E** The micrographs represent RSC96 cells dosed with H_2_O_2_ alone (**B**), H_2_O_2_ and the solution eluted from empty PCL microparticles (**C**), H_2_O_2_ with 100 µM resuspended NAC (**D**), and H_2_O_2_ with 100 µM eluted NAC (**E**). * = *p* < 0.05. Scale bars represent 50 µm

The bioactivity of NAC eluted from PCL microparticles is shown in Fig. 3. The micrograph in Fig. 3B represents RSC96 cells 48 h after original plating at a density of 4000 cells, and 24 h after the addition of 60 µM H_2_O_2_. Figure 3C represents cells 48 h after original plating and 24 h after the addition of 60 µM H_2_O_2_ with eluted solution from empty microparticles. The micrographs in Fig. 3D and E represent RSC96 cells 48 h after original plating at a density of 4000 cells, and 24 h after the addition of 60 µM H_2_O_2_ and 100 µM NAC from a stock solution (Fig. 3D) or 60 µM H_2_O_2_ and 100 µM NAC eluted from microparticles (Fig. 3E). 100 µM eluted NAC was used as this was the highest concentration of NAC that could be delivered from the eluted solution from NAC microparticles in water without exceeding 5% of the total media content [25]. RSC96 cultures administered NAC (whether from a stock solution or eluted from microparticles) exhibited an increase in density compared with their respective H_2_O_2_-alone conditions. This impact was quantified by the increase in viability associated with the two 100 µM NAC conditions observed with the CCK-8 assay and analyzed by two-way ANOVA (Main effect *p* < 1 × 10^–6^). Cells dosed with H_2_O_2_ and the eluted solution from microparticles without NAC showed similar viability as compared to cells dosed with H_2_O_2_ alone (*p* = 0.91). Cells dosed with H_2_O_2_ and freshly resuspended NAC showed an increase in viability as compared to cells dosed with H_2_O_2_ and eluted NAC (*p* = 4.4 × 10^–5^).

## Discussion

### Development of a Schwann cell model of nerve injury

An important motivation in developing a cell culture model of cranial nerve crush injuries is the ability to induce incomplete functional compromise, which is a common result after iatrogenic injury to cranial nerves coursing in proximity to a surgical field. Given the shortened timeline of in vitro cell culture models, complete recovery after injury is difficult to monitor. Nonetheless, cell culture models still serve a valuable purpose in screening and identifying potentially therapeutic agents. A model of Schwann cell injury with H_2_O_2_ was created to model the oxidative stress seen after nerve traction, compression, and crush injuries [16, 26]. While limited in its ability to comprehensively replicate the complexities associated with in vivo neuronal crush injuries, H_2_O_2_ resulted in a reproducible insult in cell culture that was utilized to screen potentially therapeutic agents at our LC_50_ concentration.

### Screening assay

Experiments with melatonin, resveratrol, and 4-aminopyridine did not show an increase in viability with or without H_2_O_2_ injury. This suggests that these agents may not impact Schwann cell recovery. NAC was observed to increase Schwann cell viability after co-dosing cells with 100 µM and H_2_O_2_ as compared to conditions dosed with H_2_O_2_ alone. Furthermore, NAC alone did not demonstrate toxicity in this assay, a desirable property when considering that local delivery often requires high initial concentrations to attain sustained therapy.

### Assessing the bioactivity of *N*-acetylcysteine delivered from sustained-release polycaprolactone microparticles

As the ideal time period for delivery of NAC after a nerve injury is unknown, microparticles with the longest sustained-release profile of NAC were selected for this application. Of microparticles synthesized with several dissolvable biocompatible polymers, polycaprolactone was the polymer found to secrete the highest concentrations of NAC for the longest period of time [23, 25]. Concentrations of 100 µM NAC or greater could be obtained from polycaprolactone microparticles for up to 7 days. The NAC released from these microparticles was thus used to compare the bioactivity of microparticle-delivered (eluted) NAC to that of freshly resuspended NAC. It is possible that high concentrations of a rehabilitative agent such as NAC for the first week after injury may be sufficient to expedite recovery in vivo. The ideal volume in which to incorporate a therapeutic concentration of NAC in vivo is also not known, but our eluted solutions of 1.5 mL are a reasonable approximation of a volume that could be easily applied to a nerve as a local injection in future in vivo studies.

### Bioactivity assessment

NAC eluted from microparticles continued to improve RSC96 cell viability at 100 µM, though Schwann cells co-dosed with resuspended NAC and H_2_O_2_ had increased viability as compared to those dosed with eluted NAC. This suggests eluted NAC may not promote viability as much as resuspended NAC, but it is still encouraging that an increase in viability was observed with eluted NAC as compared to cells dosed with an insult of H_2_O_2_ alone.

## Conclusion

There is a need for targeted therapeutics to expedite the restoration of cranial nerve function after injury during surgery. As it is typically unknown whether a nerve will function abnormally at the time of surgery, we developed a sustained-release therapeutic agent to promote Schwann cell viability which could be further investigated as a prophylactic intraoperative measure to reduce the time patients are left with functional deficits of cranial nerves after procedures. To identify a therapeutic agent, we designed a model of RSC96 Schwann cell injury with H_2_O_2_ and used this to screen different agents for their ability to promote viability. We identified *N*-acetylcysteine as a promising therapeutic agent and describe a method of delivering biologically active *N*-acetylcysteine from sustained-release biocompatible microparticles.

## Data Availability

Data is available upon request.
